# ‘Sweating Blood’: Patients' Dirty Work in Informal Complaint Biographies

**DOI:** 10.1111/1467-9566.70167

**Published:** 2026-03-08

**Authors:** Jelmer Brüggemann, Lisa Guntram, Ann‐Charlotte Nedlund

**Affiliations:** ^1^ Department of Thematic Studies—Technology and Social Change Linköping University Linköping Sweden; ^2^ Department of Health, Medicine and Caring Sciences Linköping University Linköping Sweden

## Abstract

Every day patients make informal complaints directly to care professionals. Although common in care encounters, the practice remains understudied. In this study, we focus on informal complaints through an analysis of interviews with 19 patients in Sweden, many living with chronic illness. We conceptualise these complaints as ‘informal complaint biographies’: not merely verbal expressions, but interwoven with people's lives, identities and care trajectories. As a lens through which to examine informal complaining, this study investigates ‘dirty work’: work that is considered a nuisance or even humiliating. We identify three different kinds of dirty work. ‘Disgusting’ refers to work with physical dirt entangled with the complaint. ‘Drudging’ describes patients' persistence, their effort to make healthcare function. Finally, ‘disrupting’ characterises work that challenges expertise from a vulnerable position. Our analysis shows how informal complaining can involve various types of undignifying work that cannot be separated from the complaint itself. By including work done by others than the traditional ‘worker’, namely patients, this study expands scholarship on dirty work. The study calls attention to how dirty work is shaped by systemic inefficiencies in healthcare and contributes new perspectives to complaints, research and policy, which is often wiped clean from any kind of dirt.

## 1 Introduction

Patients work hard. If they have a chronic illness they have to manage their care routines, medicines and health technologies, often while managing their own caring roles (Strauss et al. 1982; Corbin and Strauss 1985). Patients also increasingly engage in work that evaluates and aims to improve care practices (Marzban et al. 2022). With the new public management ethos endorsed in many healthcare systems, patients are increasingly encouraged to switch care providers if they believe their services are failing (Brüggemann 2017). In addition to these ‘exit’ strategies (Hirschman 1970), patients are also urged to voice their concerns, for example through online feedback forms or patient complaints.

Because patient complaints are considered an important source of ‘customer information’ about patient satisfaction and quality of care, how they can be collected, analysed and acted upon has been the subject of considerable research (Mirzoev and Kane 2018). This research is typically concerned with barriers to the use of patient complaints for care quality improvement purposes (De Vos et al. 2018) and almost exclusively deals with *formal complaints*. Much less is known about so‐called *informal complaints*, ‘made to front‐line employees or other organisational entities not through written means or channels formally designated for processing complaints’ (Gal and Doron 2007, 158), which are central to this article.

Research on informal complaints has shown that it is common (Gal and Doron 2007) and ‘can embody everything from a straightforward ‘logical’ response to instances of sub‐standard care or clinical errors, to poor communication, to a much less easily quantifiable emotional response to ‘something’ in and/or outside the health care setting.’ (Allan et al. 2015, 2112). In line with work by Winance et al. (2018), we have previously shown that patients attempt to tailor and package their informal complaint in ways that align with what they expect to be accepted as valid within care practices (Brüggemann et al. 2025). Considering the medical relevance of a concern was one dimension of patients' and professionals' work on the boundaries of ‘whining’ — complaining that is seen as to‐be‐avoided. In addition to concerns about relevance, our study highlighted a dimension through which informal complaining related to a certain type of complainer identity—being ‘whiners’. This condition was exacerbated by the temporal dimension of this work, highlighting the timing and repetition of a given complaint. Aligning with earlier work (Adams et al. 2018), complaints were almost always characterised as problematic and disruptive, urging further research into the troubles and difficulties of informal complaining practices.

Doing so, in this article and in line with our own and others' previous research (Brüggemann et al. 2025; Lenton et al. 2024), we draw on Ahmed's work on complaints (2021) in which she teases out the complexities, situatedness and non‐linearity of complaints. Building on Ahmed's *complaint biographies*, we use the idea of an *informal complaint biography* to capture how patients' accounts of their complaints are not just about the expression of concern. As Ahmed points out, complaints are not simply an endpoint or an outcome. They are interwoven with patients' bigger stories and demonstrate the extent to which complaining shapes people's lives, identities and care trajectories. And, Ahmed states, complaining involves work—at times a necessary, painful and risky labour.

In this study we examine patients' informal complaint biographies through the lens of ‘dirty work’, a concept primarily used in the sociology of work to describe work that is considered a nuisance, low prestige or even humiliating (Hughes 1958; McMurray and Ward 2014; Galazka and Wallace 2023). We ask—What kind of dirty work is expressed in patients' informal complaint biographies and how is this entangled with their lives, identities and care trajectories? We work with this research question through an analysis of interviews with patients in Sweden. Theoretically, we bring together scholarship from medical sociology about ‘patient work’ and scholarship about ‘dirty work’.

## 2 Theoretical Framework

### 2.1 Patient Work

The efforts that patients make to care for their bodies, take their medications and manage their illness trajectories, as Strauss et al. (1982) have argued, deserve to be seen as *work*. If patients' efforts are viewed only as ‘instances of good cooperation or as flowing from morally responsible persons or from nice personalities’, it is easy to be ‘less than appreciative of patients’ labours.’ (Strauss et al. 1982, 977). Since this reconceiving of the patient's role, social science research has become increasingly interested in the oftentimes invisible work that patients do to make care practices work and to better manage both their own health and the health of others (Yin et al. 2020).

Studies of people with chronic illness have shown that these patients try to manage their care in many ways (Mol 2008; Skovgaard et al. 2024). Of relevance to the current study is patient work done in the evaluation and improvement of their care practices. Strauss et al. (1982) suggest that this work can be typologised as *necessary* work of monitoring for errors or acts of *rectification*, where patients attempt to correct or report mistakes. This kind of work, although not always conceptualised as such, has mainly been studied as an aspect of patient safety. The patient’s work of monitoring and rectifying has mostly been understood as ‘speaking up’ or ‘blowing the whistle’ when they witness a safety hazard, such as medication errors or poor hygiene practices (Sutton et al. 2015).

Research into this type of patient work shows that it is fraught with problems. Many patients are afraid to confront care professionals or to be seen as a ‘bad’ or ‘difficult’ patient (Brüggemann et al. 2023; Sutton et al. 2023). However, as Sutton et al. (2023) have argued, even if they do not always express themselves verbally, they engage in many other types of work to keep themselves safe. This ‘risk work’, as they call it, is not simply rectifying and monitoring on purpose. It can also be to seek reassurance, to ensure positive relationships with care professionals and be seen as a cooperative patient. Such work tends to be invisible to care professionals and to the overall organisation of healthcare; it can also easily be mistaken for passivity.

A patient work perspective, hence, helps direct analytical attention to the efforts that patients make to not only manage their illness, but also their and others' care trajectories, even though these efforts are seldom seen or visible as work in the overall organisation of care (Strauss et al. 1982). In this article, we zoom in on one kind of patient work—their ‘dirty’ work.

### 2.2 Dirty Work

Strauss' concept of patient work aligns well with Hughes' (1958) earlier conceptualisations of work as the product of social interaction between a plethora of actors. For Hughes, in medicine, patients are also included in the overall division of labour, including the distribution and delegation of what he calls *dirty work*.

In any occupation, people perform a variety of tasks, some of them approaching more closely the idea or symbolic work of the profession than others. Some tasks are considered nuisances and impositions or even dirty work—physically, socially or morally beneath the dignity of the profession.

(Hughes 1958, 121–122)

Applied to the context of healthcare, work as a health professional is full of dirty tasks. Working in healthcare can mean the physical dirty work of caring for people who cannot take care of their own hygiene (Meldgaard Hansen 2016). It can mean work carried out near people's genitals (Bolton 2005) or the cleaning of dirty hospital environments (Stournara 2022). Social dirty work can imply interactions with ‘stigmatised’ people, such as physicians working with patients with mental illnesses or suffering from alcoholism (Strong 1980; Shaw 2004). Moral dirty work can be used to describe work with medically contested practices such as assisted death or abortion (Ward 2021; Buchbinder 2022).

Expanding Hughes' original typology, McMurray and Ward (2014) have added the category of emotional dirty work as one which is distinct from, yet often overlaps with physical, social and moral dirty work. Emotional dirt is the expression of feelings that ‘threaten the solidarity, self‐conception or preferred orders of a given individual or community’ (McMurray and Ward 2014, 1134). Using the example of the Samaritans, a UK charity where volunteers answer phone calls from people in emotional distress or with suicidal thoughts, McMurray and Ward argue that listening and supporting callers is a form of emotional dirty work. Volunteers are required to deal with a range of threatening, unallowed and out‐of‐place emotions. In healthcare, customer service workers or administrators do similar emotional dirty work. The front‐desk nature of their job means that they regularly face negative emotions, distress, anger or disgust (Whiley and Grandy 2022). Whiley and Grandy argue that these health service workers function as a shield, protecting the organisation from patients' emotional dirt by absorbing it in their bodies. Such dirty work has become necessary for the efficient functioning of healthcare organisations but has come at an embodied cost for those who absorb the emotional dirt (Whiley and Grandy 2022).

By bringing together literature on dirty work and patient work, our study is able to make three contributions. First, conceiving informal complaining as work (Strauss et al. 1982) allows us to be appreciative of the efforts patients make in situations with restricted agency and while knowing that complaining may not always be appreciated by care professionals (Brüggemann et al. 2023). Second, our study stretches the concept of dirty work beyond questions of traditional ‘workers’, that is, those who perform tasks as part of an occupation, expanding recent work that has argued for a more profound understanding of the role of ‘clients’ in professionals' dirty work. Galazka and Wallace (2023) argue for a relational understanding of dirty work, acknowledging the contributions of clients to workers' dirty work. In their conceptualisation, the dirty work is still always done by the worker, never the client. Our study contributes with a new dimension to dirty work scholarship, highlighting dirty work that is done by those traditionally seen as ‘clients’. Third, conceptualising informal complaining as also involving patients' dirty work brings new theoretical perspectives to the study of patient complaints. A dirty work perspective highlights undignifying tasks patients perform as part of informal complaining, which allows for analysis of complexities and contingencies beyond verbal expressions of concern.

## 3 Methods

This qualitative study builds on semi‐structured interviews conducted with 19 patients as its method. These interviews were part of a larger project (approved by the Swedish Ethical Review Authority, no. 2022‐02526‐01) that also included focus groups with care professionals, described elsewhere (Brüggemann et al. 2025).

During 2022 and 2023, we recruited patients through patient organisation newsletters and social media sites in Sweden. The invitation to participate explained that the interviews would broadly cover patients' verbal expressions of criticism. The only criteria for inclusion was being able to communicate in Swedish. Thirty‐nine people showed interest in the study and in the end, 19 people agreed to participate. Since we did not target a specific patient group, participants had a range of healthcare experiences. Most participants, however, lived with chronic illness and many had experiences of healthcare situations involving relatives and close ones. Five participants were men and 14 were women. Eight participants were between the age of 30 and 60, 11 were over the age of 60.

Each participant was interviewed individually by one of the researchers. Held mostly in the participants' homes or at the university, interviews lasted on average 60 min. One interview was held digitally. All participants signed an informed consent form before the start of the interview. Each interview followed a thematic interview guide which covered participants' main contact with the healthcare system, their views on patient involvement, their experience of expressing criticism or informally complaining in care situations, the expectations they felt were placed on patients and care professionals in these situations, any formal complaint procedures in which they had taken part and any connections their complaints had had to improvement of their care quality. All interviews were recorded, transcribed verbatim and names were replaced by pseudonyms.

We analysed the interview material using thematic analysis (Braun and Clarke 2006, 2014). After writing an article partially based on this material (Brüggemann et al. 2025), we returned to the analytic maps we had created. We honed in on the many efforts that patients described as dirty and difficult that intertwined with their accounts of informal complaints as they lived with illness and interacted with healthcare. We then turned our attention to dirty work literature. We moved individually back and forth between our codings and this literature—in particular conceptualizations of different kinds of dirt—and together constructed new analytic maps and theoretical connections. We focused on different aspects of patients individually managing dirty efforts as well as their relation to structural dimensions and deficiencies of the healthcare system—a reflexive process, also informed by our previous research (Brüggemann et al. 2023, 2025) and our knowledge of the Swedish healthcare system. Through this abductive process we identified three themes, each highlighting one dimension of patient’ dirty work, below presented as *disgusting* (informed by sub‐themes such as ‘cleaning physical dirt’ and ‘managing bodily fluids’), *drudging* (informed by sub‐themes such as ‘coordinating efforts’ and ‘energy demanding persistency’) and *disrupting* (informed by sub‐themes such as ‘questioning expertise’ and ‘being non‐compliant’). In the process of delineating new analytic and theoretical connections we used ‘dirty work’ as an analytic lens through which we could study informal complaint biographies. Using dirty work as a lens does not necessarily imply that our participants experienced their informal complaint practices as dirty work. Neither does it exclude that these practices also can give meaning and a sense of agency and satisfaction (see e.g. Galazka and Wallace 2023). Rather, it enabled us to highlight particular dimensions of patients' informal complaint biographies and how there entangled with their lives, identities and care trajectories.

Our empirical material is situated in a Swedish healthcare context. Nationally regulated but locally managed and administrated, Swedish healthcare is primarily tax‐funded (Janlöv et al. 2023). Increasingly, Swedish healthcare governance is dominated by ideas of New public management and patients are increasingly positioned as customers (Hasselbladh et al. 2008). Recent reforms of the Swedish healthcare system have therefore prioritised increased patient choice as the mechanism driving improved quality and availability of care (Janlöv et al. 2023). Along this decline of the traditional Swedish welfare state, patients are—through extended patient rights—assigned increased responsibilities for their care choices and trajectories. Regarding formal complaints, patients are expected, initially, to file their complaint with the relevant care organisation for redress. Only if still unsatisfied can they complain directly to the central Health and Social Care Inspectorate. The exact chain of formal complaint handling looks different in each of Sweden's 21 regions (Janlöv et al. 2023).

## 4 Analysis

In our analysis, we have identified three kinds of dirty work expressed through patients' accounts of informal complaints. Taken together, these kinds of dirty work show how complaining is entangled with people's lives, identities and care trajectories and how informal complaining can involve unworthy work that cannot be separated from the complaint itself.

### 4.1 Disgusting

Gooey, smelly, messy. Scholarship on dirty work in healthcare settings has studied the many ways in which care professionals deal with *physical taint*. Professionals work with patients who cannot manage their own hygiene or they work close to people's genitals (Bolton 2005; Meldgaard Hansen 2016), as previously mentioned. There are many examples of physical dirt present in the everyday work of care professionals: out‐of‐place bodily fluids, disconnected body parts and organs or pungent odours. Our analysis of patients' informal complaint biographies identified recurring examples of *disgusting* tasks: patients having to carry out physical dirty work — dealing with blood, sweat or urine, for example — as part of their care regimen.

Karin's experience with the healthcare system can be described as an account of the dirty work she was obliged to perform as the result of the dysfunctional organisation of her care. She described the disgusting work she had to do when her nephrostomy started to leak. Installed after her cancer surgery some years ago, the thin catheter tube and bag protruded from her abdomen and was used to collect urine.

And when it’s been there for a while you start to smell like urine, which I of course don’t want to do. It all started this summer. They’d given me a new kind of connection, from the kidney. I fought them over this one and told them, “This doesn’t work for me, I can’t live like this”. I keep washing loads of clothes and feel bad about it mentally, constantly having to check if I am wet or not.

Karin brought this issue to the attention of care professionals at her primary care centre, but they could not give her the right type of connection because it was not a ’standard’ piece of equipment that was regularly procured—a clear misfit between her individual needs and the logics of standardisation central to the Swedish healthcare system (Brüggemann et al. 2023). Instead, when visiting the hospital to have her nephrostomy fitting changed, Karin asked if she could have a better‐fitting connection. She expressed her frustration with the fitting and she had described the discomfort she felt smelling urine, risking infection and constant washing. Dealing with the smell of urine entailed additional psychological and physical work for Karin. The dirty work underpinning her complaint and her request for a more suitable connector, is more than just the origin of a typical complaint trajectory moving in a straight line from concern to redress (Mulcahy and Tritter 1998). Karin's expressions of concern about the tubes and the smell cannot be isolated from the embodied dirtiness Karin describes she carries with her. Her dirty work is not the starting point of the complaint—it is part of her informal complaint biography. A biography about organisational failure and the physical taint it created.

Jonathan presents a contrasting account. Living with chronic illness, he often chooses to perform disgusting tasks rather than risking the consequences of being seen as a ‘difficult patient’ if he informally complained (Brüggemann et al. 2023). During his interview, Jonathan spoke about a situation which emerged after his surgery.

The cleaning was really not that great and I was kind of worried about it because of my low immune system. I remember there was blood on the bed, like on the metal bedframe and the floors were dirty and everything and it’s not really the kind of thing you want to be worried about when you feel that bad. It was disgusting in there.

Researcher: Was that a concern you felt you could raise or how did you deal with it?

Well […] I didn’t have the energy, no, so I took disinfectant and cleaned a lot myself. I didn’t want to, but I also don’t want people to …I don’t want to be a burden.

As we have previously argued (Brüggemann et al. 2025), strong norms exist around informal complaining. Patients not willing to cross the boundary into ‘bad’ informal complaining may instead choose to keep silent. As Jonathan's account shows, this does not mean that patients are necessarily passive. Despite his low immune system, Jonathan cleaned the old blood off his bed, a disgusting and potentially dangerous task. Patients doing such dirty work tells us something about the difficult character of informal complaining in healthcare settings. With the risks and uncertainties associated with informal complaining, non‐complaint can also be read as part of an informal complaint biography, as Ahmed (2021) has argued. Jonathan's decision to clean the bed, his ‘choice’ of non‐complaint over non‐compliance, illustrates the efforts that some patients make to get healthcare to work for them while absorbing the stigma associated with being seen as difficult and disruptive patients.

When Mona was faced with a dirty hospital environment, she ended up in a similar situation. For some time, she had been committed to a hospital's compulsory psychiatric care ward, along with about 20 other patients.

I am pretty tidy myself and it was really nasty that place, with very bad cleaning on the ward. So I went and cleaned it up. There was blood and faeces, all kinds of stuff, but I cleaned it up because I thought it was humiliating. A nurse told me to “write to the top management” and this was during a time when collective labour agreements were being re‐negotiated, so it was really important what the cleaners did, what the auxiliary nurses did and what the nurses did, so [the nurses] refused to clean. […] So they closed them and we needed to share one bathroom and it was unsustainable, so that’s why I cleaned up.

Mona's example illustrates a tendency on the part of many professions to transfer to other professions what they perceive as dirty work. Nurses pushed the work of cleaning the toilets on to the cleaners and, by effect, also on to the patients. Rather than helping her, the nurse told Mona to complain to senior management, thus implicitly agreeing that this was a complaint‐worthy situation and a humiliating task for patients to carry out, pushing back against the management. Although we acknowledge the importance of power dimensions in this situation, Mona's account is a striking example of how systemic failures create new dirt in healthcare practices for care professionals and patients to deal with—a kind of ‘pollution’ by the healthcare system.

A final form of physical dirty work in patients' informal complaint biographies highlights how patients' disgusting tasks can amplify the implications of their complaints and the severity of their condition. Rita talked about her stay in hospital when she had fallen off a horse and the situations that took place when care professionals considered sending her home early.

I sat up, because they were about to come for the round visit and I sat myself in an armchair. Anyway, I had access to a walking table, with a small motor, that I could use to get up and go the bathroom. So, I stood there in the middle of the room, with some staff in the room and I completely wet myself, it just ran. At that point they came for their round visit and I had just found out that they were about to discharge me, they had talked about it at least. So, they opened the door [laughs] and there were medical students as well and “oops” they said and closed the door. “No, just come in”, I said, “please, this is what you are about to send home, this is what I look like, please have a good look” and they thought it was really challenging.

To show her objection to the proposed decision to send her home, Rita raised her voice and forced care professionals to witness her physical condition. Her spontaneous informal complaint was amplified by the physical aspect of her protest. In this situation, Rita mobilises the physical dirty work as a way of enacting a form of vulnerability, using the shameful leaking of her body to create an affective response on the part of the care professionals and force them to reconsider their decision. Whether or not the wetting itself is seen as a form of work, the key point is the reminder of the work Rita must do to ensure her urinary incontinence is managed properly, thereby making sure she fits the social order on the ward. This situation and its enactments of vulnerability cannot be understood by merely looking at Rita's verbal expressions of concern in that moment. Her outburst was intrinsically related to her incontinence, making care professionals feel and demonstrate accountability for her near future should she be discharged too soon. Her dirty work functioned as a form of resistance, altering her care trajectory.

### 4.2 Drudging

Patients often spoke in very general terms about the struggles they had with health care contacts. In these accounts, they spoke of complaining as an ongoing effort to get the care and support that they themselves or their loved ones needed. Rita mentioned,

There was no other alternative. I was probably a real pain in the ass in all ways possible, you know. I was not rude to the staff, that I was not. […] I fought like an animal; writing, calling and went on and on.

These efforts often continued over a long period of time, which typically made them invisible yet at the same time essential to make care practices work (Corbin and Strauss 1985). Informal complaining in this way—ill‐defined dissatisfaction and low‐grade resistance to the care system, as Ahmed (2021) points out, cannot be confined to a single event in time or even to several consecutive events. This discourse has become intrinsically intertwined with their own or their loved ones' life and care trajectories. The time‐consuming efforts to ensure needs are met, the continued onslaught of scheduling appointments, getting test results, coordinating contacts with and between care providers, while attempting to remember to ask questions or raise concerns becomes a form of drudgery. Drudging could be overwhelming, the patients stressed, because these tasks needed to be managed alongside the chores connected to everyday life and one's illness itself. Despite the drudgery being a burden to them, patients understood these tasks were too urgent to disregard.

The dirtiness of drudging was explicit in the patients' lingering frustrations over the efforts involved in coordinating within and between different care facilities. Thea, for example, recalled a time when she had gotten so angry that she had called a patient advocate, who in turn contacted the clinic in question.

Then [the patient advocate] called the manager at the [clinic] and he said “You can come right away”. But I told him, how was I supposed to do that — to get there right away — when I’m at work? You can’t stay home all day because you can’t get through to the clinic. You have to go to work. Even if you call in the morning you may not even get through to them at all. Not that long ago I called them at 08.05 and they said that their daily quota of calls had already been reached. “Please call us again tomorrow”. I think that’s crazy!

Thea exemplifies a common frustration over imbalanced responsibilities within the healthcare system. Thea is expressing the feeling of having to care *too* much about things that ought to be taken care of by the healthcare system. This echoes discussions in the patient involvement literature, where questions are raised to what extent patients should be responsible for monitoring their illness and identifying any risks associated with their care (Sutton et al. 2015). Patients struggling with timely access to care—a contemporary challenge in Swedish healthcare– provides yet another example of how structural inefficiencies pollute the healthcare system, demanding patients to engage in a drudging type of dirty work.

Raising concerns directly with the responsible healthcare provider was seen by patients as a sensitive and delicate task, to be managed on top of everything else. It required emotional investment and careful consideration of the potential consequences. At the end of the day, many patients simply did not have the strength to complain when struggling with their illness. When Magdalena was asked to describe what patients could do to express concerns in health care encounters, she replied,

It is very difficult when you’re also depending on healthcare and I just didn’t have the strength. My god, I was just trying to make it through the day. I could not even manage to take a shower, although I was working. It was that bad. In such situations you simply have no strength.

In contrast, Jakob's struggles were not with illness but rather struggles to remove an incorrect diagnosis from his medical records. Jakob explained how his care provider told him that they ‘did not like to remove records’ and that removing the diagnosis would ‘take a long time and would require a lot of administrative work’. Eventually, a note was added to Jakob's record to show that the diagnosis was no longer valid. However, since this note was not always obvious to other healthcare providers when they accessed his records, the diagnosis still had serious implications. For example, one time when seeking psychological support Jakob had been denied care, a rejection which had a negative impact on him at an existential level.

It was hard. I felt for a very long time that I wasn’t human, I can tell you. I felt that I’m not…I don’t belong to society, I don’t belong to the people, I don’t belong to my neighbours…. You know, they treated me differently, so I felt like an alien. I did not belong, I thought I was a lower human.

Jakob's dirty work was the drudgery of trying to clear his false diagnosis. The systemic obstacles, the begrudging compromise of adding a note to his file affected not only Jakob's interactions with the healthcare system. It also had an impact on his sense of belonging and sense of self—an example of how drudgery can come with an embodied cost when absorbed by patients or relatives who need to engage in such dirty work (Whiley and Grandy 2022).

Previous research has suggested that dirty work can be discursively reframed so that the means and purpose of certain occupations is understood in more positive terms (Ashforth and Kreiner 2013). In the interviews, patients repeatedly tried to emphasise the value of their drudging work: it was enacted to make life liveable for themselves or for their relatives. It was intended to create better conditions for future patients, reframing dirty and stigmatised tasks as something valuable (Ashforth and Kreiner 1999) and necessary. As Rita explained, at times the healthcare system made it impossible to even offer suggestions for improvements which might make things better. Given that the only other option in these cases is far worse — as we have discussed elsewhere, resigning yourself to your own death or the death of your loved ones (Brüggemann et al. 2025) — there is simply no other alternative but to keep on with the dirty work.

Drudgery was an integrated element of many patients' informal complaint biographies. Patients described ongoing struggles with the healthcare system and the dirt it created, characterised by persistent efforts to coordinate, manage and re‐direct care trajectories. This is not only a delicate balance act for patients, but at times also undignifying work in the light of suffering, vulnerability and dependencies brought about by illness.

### 4.3 Disrupting

The third type of dirty work which we identified as part of informal complaint biographies is disruption—the undignifying work involved in having to challenge the healthcare system's normative order and power relations. This work is mainly connected to emotional dirt (McMurray and Ward 2014)– the oftentimes negative feelings associated with actions that threaten a preferred order.

When Stefan was diagnosed with prostate cancer, at one of his appointments the oncologist suggested that when metastases were discovered castration might be a possible treatment. Stefan was very critical of this doctor's opinion,

This oncologist, he was rude and unprepared and treated me like this 75‐year‐old “oldster”, like a bit nonchalant, as if to say just give him some bicalutamide and he’ll die in a few years. And in my medical record he wrote that radiation therapy would not prolong the patient’s life. You can’t really write that in a medical record. Well, anyhow, I got annoyed and asked for a second visit.

At the second visit the oncologist confirmed his original treatment proposal. Stefan then sought a second opinion from an oncologist at another hospital, who largely confirmed the initial assessment but recommended new imaging. This recommendation led to a new scan which showed reduced metastases and opened the way for other treatment options. In the multidisciplinary team meeting (MDT), where Stefan's treatment would be decided, he continued to challenge the physicians' agreed opinion. Stefan wanted the MDT to consider treatments that were not common practice in Sweden nor mentioned in treatment guidelines. He gathered evidence for his position through a literature review on Pubmed, which demonstrated the benefits of a specific kind of radiation for patients in Stefan's position. At the MDT meeting, this alternative treatment was discussed and approved.

Stefan's efforts can be understood as a form of disruption. He persistently challenged the experts in the field by seeking alternative sources of authority and mobilising his biomedical knowledge. In so doing he strengthened the medical validity of his complaint (Brüggemann et al. 2025). Feeling forced to question dominant order, we argue, can be humiliating if done from a vulnerable position and without being taken seriously. Although Stefan displayed no anxiety in verbalising his thoughts to external authorities, a marker of strong cultural health capital (Shim 2010), others might be all too aware of their vulnerable position and the emotionally charged nature of any encounter in this context. Informal complaining, as Stefan's account illustrates, is as closely connected to his previous education and work‐life experience as it is to his current care trajectory.

For Karin, in a similar vein, challenging care professionals' decisions was ‘an everlasting battle’. After being returned to the care of her GP after specialist spinal care, the GP said she needed an injection due to vertebral complications. When asked why she needed the injections, the GP could not give her a reasonable answer. Karin then contacted the specialist spinal unit to see if she could talk to the specialist who had seemingly prescribed the injections. However, Karin was only able to speak with a nurse.

“Well, you need to take this injection because you have vertebral compressions”. “Yes, but I want to know if I my back can get worse, can I get more vertebral compressions? I want to know that.” “Yes, I understand but probably no one can answer that.” “Probably no one? But the person who made the decision that I need this injection must still have had a…” So I kept on like this. “I want to talk to that doctor!” “But we don't have that policy here.” “No, maybe you don't”, I said, “but my GP can't answer this, so I want to talk to the doctor who made the decision! That person must be an expert.” And after half an hour, this person says “Well, you seem very persistent, so I guess I'll book you a phone appointment”. “Yes, thank you”, I said.

Karin's and Stefan's accounts, we argue, show how the dirtiness of informal complaints is manifested in questioning those in power from a disadvantaged position, in disrupting dominant power structures. Patients such as Karin are aware of the risk they take in crossing this invisible line of ‘acceptable’ and coming to bear the stigma of a ‘difficult patient’ (Brüggemann et al. 2023). Karin knew

that you should do as the doctor says […], but it was like, “You can do as you like, it's your decision”. Yes, I understand that, but I still need tools to be able to make this decision. Is it better for me to wait or is it life‐threatening for me to wait? But no then it becomes, then it becomes like “well, you are a difficult patient”.

Jonathan's account, however, shows that disruption is not always possible. The dirty work of disrupting, such as that of drudging, is particularly difficult when the patient is sick and hospitalised. Jonathan describes how at one point his medical prognosis was very poor and the doctors wanted to test a new medicine that was still under trial.

I read through and didn't really want to take it because I was afraid of the side effects […], there were other medications that were approved and ready for me, but they wanted to try a new kind […] there was also an occasion where—where I was argued against. I mean, I was persuaded to take it in the end. I remember that I actually cried then [laughs]. First I tried to hold out with the nurse and just said but I absolutely don't want to take this, it doesn't feel right and she calmed me down. But then she went out and talked to the doctor who came in and, yeah, talked maybe for half an hour and in the end, he persuaded me to take it.

Jonathan described feeling weak and like a ‘guinea pig’ or a lab rat. Rather than making a sustained disruption, however, he was only able to make a short‐lived ripple. He tried to say ‘no’ and to challenge the power of the care professionals but lacked capacity to do so.

Finally, patients also provided examples where their disruption was directed towards a normative orientation within the healthcare system which questioned the legitimacy of their illness. Linn described the disruption which took place when two care clinics could not come to an agreement on her sick leave while she was pregnant.

And they didn't read through my entire medical record and didn't see all the diagnoses or the reasons behind, like, for the pain issues and everything. […] Yes, I had to push them on several occasions, both at the care centre and with my midwife, then… I had to push them and ask “What is the problem?” because you can enter the digital platform and read your own medical record and notes and see what they write to each other. And I got quite…very irritated, because they blamed each other that the responsibility was on the other.

When Linn described her experiences, she said more about her medical condition, a chronic autoimmune disease that affects mostly women and whose symptoms are still highly contested. Linn felt that her need for more care than an ordinary pregnancy and delivery would require was disregarded because medical norms about women's health meant her illness was not taken seriously. The dirty work in Linn's informal complaint biography suggests that the disruption of complaining can also be directed at grander societal discourses and social stigmas—a kind of stigmatised norm‐breaking work also shown in other contexts (e.g., Darakchi and Valkovičová 2025).

As this section has shown, the dirty work of disrupting can take various forms by challenging normative orders and power relations. This work is not only difficult but can indeed be humiliating and dirty if vulnerable patients' questioning is seen as problematic non‐compliance.

## 5 Discussion

In this article we have studied patients' informal complaining through a lens of dirty work. This lens helped us capture how informal complaining, in patients' accounts, is more than an actual expression of concern or critique. Mobilising Ahmed's concept of the complaint biography (2021), we highlighted that informal complaining requires patients to engage in unworthy and unpleasant work. This stands in stark contrast to dominant perspectives on patient complaints, especially formal complaints, which suggests they have been wiped clean of this kind of dirt (Mulcahy and Tritter 1998). Our participants provided rich accounts of the disgusting, drudging and disruptive tasks that shaped their informal complaint biographies. Their work involved more than a desire to express a concern or raise a critique. Although conceptualised as dirty work, it was rooted in patients' sense of self and human dignity and aimed at promoting the best healthcare options and improving conditions for future patients. The sociology of work and its related concept of dirty work has enabled us to say more about the sociology of complaint.

Applying dirty work scholarship to the understanding of patients' informal complaint biographies makes an important leap. Galazka and Wallace (2023) and Neal (2018) have each pointed out that dirty work can be understood in relational terms, as a task shared between the worker and the ‘client’. Like Galazka and Wallace, who claim that ‘scholars have long under‐appreciated clients’ contribution to workers' experience of work and stigma’ (Galazka and Wallace 2023, 719), our research builds on the idea that patients' participation in healthcare can also be viewed as a form of work. Whereas a focus on patient accounts does not provide insights into how care professionals directly shape patients' dirty work, our study contributes to relational understandings of dirty work by analysing work from a ‘client’ perspective and their views on this work as done in relation to care professionals and healthcare structures. When doing so, we have highlighted patients' dirty work in informal complaint biographies yet not linked this to an identity of ‘dirty workers’. Rather than assigning dirty work to specific roles, we have demonstrated the analytic value of studying patient accounts of their work and how that work is linked with their lives and care trajectories.

Connecting back to a typology of patient work by Strauss et al. (1982), we see patients doing different types of work in their informal complaint biographies, each with different implications for the dirt and stigma patients have to deal with. If patient work *substitutes* for dirty work that could have been done by staff, then potential stigma is transferred from the professional to the patient. An example would be patients' accounts of cleaning dirty toilets or bloody beds, work that typically would have been done by staff. Work that patients deem *necessary* to safeguard care or that *rectifies* staff or systemic errors, however, does normally not involve any transfer of dirt from the professional to the patient, as this is patient work done in relation or reaction to the healthcare system or work by staff. In cases of such disgusting, drudging or disruptive patient work, dirt may transfer from the work to patients' identity. An example would be patients' accounts of relating drudging or disruptive work to an identity of the ‘difficult patient’. Monitoring care providers or questioning their decisions were actions described as necessary for patients yet crossing boundaries of what would be perceived as acceptable and compliant patient behaviour (Brüggemann et al. 2023). Although we underscore the importance of power and relational dimensions to patients' dirty work, we have also highlighted how transfers and redistributions of dirt among various healthcare actors is very much conditioned by governance structures and political discourse. In the Swedish context and beyond, new public management and increased emphasis on patient choice have radically altered relations between care professionals and patients and shifted the burden of care onto patients (Hasselbladh et al. 2008; Mol 2008). These shifts are further catalysed by understaffed healthcare organisations and technological imaginaries of self‐care and remote care (Arnelid 2025). Drawing from our analysis, we suggest such systemic developments not only imply that more dirt can be transferred from professionals to patients, but also that new dirt and dirty work may be created—a potential *pollution* of and by the healthcare system.

The central concepts used in our study—*informal complaint biographies* and *dirty work*—should here be seen in the light of the chronic illnesses with which most of our participants have lived and upon which most research on patient work builds (Corbin and Strauss 1985; Mol 2008). Although our empirical material provided us with rich insights into the entanglements between informal complaining and patient care trajectories and illness experiences, it is likely that patients with only incidental or superficial interactions with the healthcare system would have given us different accounts. It is likely that the dirty work associated with their complaints would be less tangled up with issues of identity, stigma or long‐term risk assessments. Our material is also likely shaped by the homogeneously white and fluent Swedish participants. Being reminded by Ahmed that the negative effects of complaining ‘are sticky as well as picky’ (2021, p. 149), we know that ethnicity and cultural health capital (Shim 2010) have an important impact on healthcare trajectories and outcomes and we would likely have seen accounts of other kinds of ‘systemic pollution’. The dirty work performed by some of the patients in our material would also not be available to less ‘louder’ patients without the necessary cultural, linguistic or socio‐economic resources to voice concerns or re‐direct care trajectories.

These methodological reflections beg the question of how informal complaint biographies might illustrate other ‘strategies’ patients adopt for managing their care concerns or critiques. In our material, for example, some patients described an ‘exit’ strategy (Hirschman 1970) by switching care providers. Hirschman shows that there is a trade‐off between voicing concerns and switching providers. If voicing concerns is deemed difficult, patients are more likely to switch providers if that option is available. Even though more countries are granting patients the right to choose their healthcare provider, including Sweden (Swedish Ministry of Finance 2008), in practice exercising this choice can be difficult, especially for patients who need specialised care or whose geographical location or specific case limit their options (Brüggemann 2017). However, if exit is not an option, patients' voices may increase, unless they lack the resources, opportunities or energy to voice concerns and in that case likely remain silent. We argue, like Ahmed (2021), that compliance, in this case, is an equally important part of patients' informal complaint biographies. Acquiescence may even require an additional kind of dirty work at which we have so far only hinted and which we encourage future research to explore: a submergence of concern, similar to the way frontline workers absorb complaints to protect an organisation (see Whiley and Grandy 2022). In this article, we have shown that to refrain from expressing complaints does not necessarily mean avoidance but rather absorption of stigma. Future studies could explore more specifically the strategies that patients use to manage stigma in the context of complaints and dirty work (Ashforth and Kreiner 1999). Further, patients' complaint absorption or ‘keeping quiet’, does not necessarily mean that concerns are not shared. It is common for patients to take their dissatisfaction and share it in all kinds of informal and online settings (Mulcahy and Tritter 1998; Stage et al. 2024). The particularities of patients' stigma management in relation to complaining and non‐compliance in online settings would be yet another important topic for future research.

In this study we have argued that patients, like healthcare service workers, also perform a variety of dirty, humiliating tasks that are necessary for health care practices to function or improve or for patients to get the care they need, either for themselves or their loved ones. What is significant is that these tasks are performed by people who live with or take care of people with chronic illness. It is through this vulnerability that we have considered the dirty work in patients' informal complaint biographies, a concept that enabled us to examine the ways in which complaints shape and are shaped by people's life situations and care trajectories. Our analysis has also highlighted how patients' dirty work is not necessarily transferred to them from care professionals, but can be produced and shaped by systemic inefficiencies in healthcare. These insights expand scholarship on dirty work and provide new theoretical perspectives for research on patient work in general and the sociology of complaint in particular. It has long been argued that patients need to ‘sweat blood’ to make the healthcare system work for them and their loved ones. Our study emphasises that this effort is central to informal complaining. Broadening the perspective on dirty work and nuancing the understandings of informal complaints is crucial to improving structures for patient involvement, patient feedback and care quality development.

## Author Contributions

**Jelmer Brüggemann:** writing – review and editing, writing – original draft, investigation, formal analysis, conceptualization. **Lisa Guntram:** writing – review and editing, writing – original draft, investigation, formal analysis, conceptualization. **Ann‐Charlotte Nedlund:** writing – review and editing, writing – original draft, investigation, formal analysis, conceptualization.

## Funding

This study was supported by the Swedish Research Council (Grant No. 2020‐01594).

## Ethics Statement

This study has been approved by the Swedish Ethical Review Authority (No. 2022‐02526‐01).

## Consent

The authors have nothing to report.

## Conflicts of Interest

The authors declare no conflicts of interest.

## Permission to Reproduce Material From Other Sources

The authors have nothing to report.

## Data Availability

Research data are not shared.
